# Pharmacist-led educational intervention to improve knowledge, medication adherence, and asthma control among asthma patients at Ayder Comprehensive Specialized Hospital: A protocol for randomized controlled trial

**DOI:** 10.1371/journal.pone.0349805

**Published:** 2026-07-16

**Authors:** Mekuriaw Dereje Tekele, Goitom Belay Kassa, Yainu Welegerima Welekidane, Derbew Fikadu Berhe, Abadi Kahsu Gebre, Afewerki Gebremeskel Tsadik

**Affiliations:** 1 Department of Clinical Pharmacy, School of Pharmacy, College of Medicine and Health Science, Mizan-Tepi University, Mizan-Aman, Ethiopia; 2 Department of Clinical Pharmacy, School of Pharmacy, College of Health Sciences, Mekelle University, Mekelle, Ethiopia; 3 Biomedical Science Division, and Center for Equity in Global Surgery, University of Global Health Equity, Kigali, Rwanda; 4 Nutrition & Health Innovation Research Institute, School of Medical and Health Sciences, Edith Cowan University, Perth, WA, Australia; Addis Ababa University, ETHIOPIA

## Abstract

**Objective:**

To evaluate the effect of a pharmacist‑led educational intervention on medication knowledge, adherence, and asthma control among adult asthma patients at Ayder Comprehensive Specialized Hospital, Ethiopia.

**Methods:**

A single‑blind, parallel‑group randomized controlled trial will be conducted. All eligible adult asthma patients on active follow‑up at the chest clinic of Ayder Comprehensive Specialized Hospital will be included. Participants will be randomly assigned via a computer‑generated sequence to an intervention group (structured pharmacist‑led education using teach‑back, illustrated pamphlets, a follow‑up phone call and SMS) or a control group (routine care). Outcome assessors will be blinded to group allocation. Outcomes will be measured at baseline and after three months, including medication adherence (GMAS), asthma control (ACT), medication knowledge, correct inhaler technique, and domain‑specific adherence barriers. Data will be analyzed using intention‑to‑treat with multivariable binary logistic regression, chi‑square tests, independent T-test, McNemar test, Mann‑Whitney U test, and Fisher’s exact test where appropriate. Subgroup analyses will be performed to explore whether the intervention effect differs across patient characteristics. A p‑value < 0.05 will be considered statistically significant, with 95% confidence intervals reported.

**Trial registry:**

Pan African Clinical Trials Registry (PACTR202508565637179).

## Introduction

Asthma is a persistent inflammatory condition of the respiratory tract that impacts more than 339 million individuals globally [1,2]. Although the condition is more common in wealthy nations [3], its impact is rising notably in developing countries, including Ethiopia, where the estimated prevalence among hospital attendees is 9% [4].

Effective asthma treatment is primarily built on attaining and sustaining proper symptom management [2,5]. Global clinical recommendations, such as those issued by the Global Initiative for Asthma (GINA) [2], highlight the necessity of regular controller treatments, notably inhaled corticosteroids (ICS), for decreasing airway swelling and averting acute episodes [6,7]. Nevertheless, merely prescribing these potent drugs is insufficient; their effectiveness relies fully on the correct usage by patients [8,9].

Consequently, poor adherence to prescribed medications represents a major obstacle, acknowledged as a leading cause of unsatisfactory asthma outcomes [10,11]. In the context of asthma, non‑adherence is a multifaceted issue involving faulty handling of inhaler equipment [12–14], which lowers pulmonary drug deposition [15], and irregular timing or dosing [16], where patients fail to take medicines as often as directed [17]. Research consistently reveals that non‑adherence to controller drugs can reach up to 70%, resulting in inadequate symptom control [5], more frequent emergency visits [18,19], hospital admissions [20], and preventable deaths [21]. In the Ethiopian setting, published figures place non‑adherence to inhaled therapies between 19.1% and 40.8% [4].

A marked shortage of patient understanding about the illness and its treatment often leads to unsatisfactory asthma management [22]. Numerous patients do not clearly comprehend the roles of their drugs, especially the difference between controllers and relievers [16]. Moreover, a large share of patients exhibits improper inhaler handling [17], with certain investigations reporting mistake rates over 50% [14,23]. Faulty inhaler technique is linked to below‑therapeutic drug delivery to the lungs [24], making even the strongest medicines ineffective [25]. This mix of inadequate knowledge and incorrect administration can produce a cycle of treatment failure [26], patient disappointment [27], and reduced confidence in the prescribed regimen [28,29].

Pharmacists, being among the most reachable health professionals, are ideally situated to interrupt this cycle [30,31]. Educational initiatives led by pharmacists have become a promising approach to address knowledge deficiencies and improve medication taking behavior [1,32]. Such organized programs extend beyond routine dispensing to offer thorough counselling on the nature of the disease, the purposes of different drugs, hands‑on training in inhaler use, and methods to overcome adherence barriers [30,33,34]. By focusing on the underlying causes of non‑adherence, knowledge gaps, and skill limitations, these interventions have proven successful in boosting adherence and subsequently enhancing asthma symptom control [35,36].

Although several Ethiopian studies have recorded the high frequency of poor medication knowledge, inaccurate inhaler technique, low adherence, and inadequate asthma control, evidence regarding the effectiveness of pharmacist‑led programs to remedy these problems remains scarce. Therefore, this trial aims to assess the effect of a structured, pharmacist‑led educational intervention on medication knowledge, inhaler technique, adherence, and asthma symptom control in adult asthma patients attending Ayder Comprehensive Specialized Hospital.

## Methods

### Study design

This study is a single‑blind, parallel‑group randomized controlled trial (registered with the Pan African Clinical Trials Registry; PACTR202508565637179). It is a non‑pharmacological, behavioral intervention study, with a total of 131 eligible patients enrolled (all eligible patients from the target population). Screening, intervention, and assessment were conducted at the Chest Clinic of Ayder Comprehensive Specialized Hospital (ACSH). A SPIRIT schedule of enrollment, interventions, and assessments can be found in Figure S1. This protocol has been developed following the Standard Protocol Items: Recommendations for Interventional Trials (SPIRIT) guidelines [37].

### Subject recruitment

Participants for the study were recruited from the Chest Clinic of Ayder Comprehensive Specialized Hospital (ACSH). To ensure recruitment of the target sample size, the research team consecutively screened and approached all eligible asthmatic patients attending the clinic during the recruitment period. Potential participants who met the inclusion criteria received a detailed explanation of the study’s purpose, procedures, potential benefits, and risks. Those who provided written informed consent were enrolled and randomized into the trial.

### Sample size

A formal a priori sample size calculation was not performed because the study aimed to include the entire eligible population. The final sample consisted of 131 adult asthma patients. A post‑hoc power analysis was conducted using parameters derived from published studies in low‑income countries. Based on a meta‑analysis of adherence in Ethiopian asthmatic patients, the pooled baseline adherence is approximately 40% [4]. A clinically meaningful improvement of 20 percentage points (to 60%) was considered, consistent with pharmacist‑led intervention studies in Nigeria [38] and Vietnam [1]. Using a two‑proportion test with α = 0.05 (two‑sided) and a sample size of 131 (approximately 65 per group), the study achieves 80% power to detect an absolute difference of 20 percentage points.

### Eligibility criteria

Participants will be eligible for the study if they are aged 12 years or older with a physician‑confirmed diagnosis of asthma, are currently prescribed at least one daily controller medication, have been on asthma medication for a minimum of three months prior to enrollment, are able to read and understand the educational materials (if the patient cannot read, a literate caregiver may assist, and all content will also be explained verbally), have access to a telephone for follow‑up calls and text messages, and are willing to provide written informed consent. Participants will be excluded if they are not currently prescribed a daily controller medication; are unable to read and have no literate caregiver to assist; have no telephone access; have a cognitive or severe psychiatric condition that impairs their ability to understand the study or provide consent; or are unable to communicate effectively in Tigrinya or Amharic.

### Randomization

All participants who provide written informed consent and meet the eligibility criteria will be randomly assigned in a 1:1 ratio to either the intervention group or the control group. A block randomization method will be employed using a computer‑generated random number sequence prepared by an independent investigator who is not involved in participant recruitment or intervention delivery. The allocation sequence will be concealed from the research team enrolling participants using sequentially numbered, opaque, sealed envelopes.

## Intervention

### Intervention group

Participants assigned to the intervention group will receive standard care augmented by a structured, multi‑component intervention delivered by trained post baccalaureate Doctor of Pharmacy interns under the supervision of a clinical pharmacy specialist. The intervention will begin on the day of enrollment with a 30‑minute, one‑on‑one, face‑to‑face session. This session will cover comprehensive asthma education, including common triggers and actions to take during an exacerbation, the importance of daily adherence to controller medications, and hands‑on inhaler technique training using a placebo device and the teach‑back method for immediate corrective feedback. It will also include managing medication side effects and developing personalized adherence strategies. Participants will receive illustrated educational pamphlets in the local language (Tigrigna). The initial session will be reinforced by a 2‑minute follow‑up phone call in the first month to assess their general condition, check their adherence status, and address any questions or challenges they encounter, as well as a standardized motivational text message (SMS) in the second month to encourage sustained adherence to their medications and follow‑up. Participants will also be informed that they may initiate contact with the study team at any time to address questions or concerns.

### Control group

Participants assigned to the control group will receive standard routine care. They will not receive any structured education or counseling from the research pharmacist or study team.

### Outcome measures

The following outcomes will be assessed at baseline and at three months (end of follow‑up). Medication adherence will be measured using the General Medication Adherence Scale (GMAS) [39], an 11‑item tool scored from 0 to 33; a score ≥27 will define “adherent” and <27 “non‑adherent”. The GMAS also provides domain‑specific scores for behavioral, burden‑related, and cost‑related adherence barriers. Asthma control will be assessed using the Asthma Control Test (ACT) [40], with a score 0 indicating well‑controlled asthma. Medication knowledge will be evaluated by the patient’s ability to correctly distinguish between controller and reliever medications. Inhaler technique will be assessed through direct observation using a standardized checklist adapted from National Institutes of Health (NIH) guidelines, recorded as correct or incorrect.

### Data collection

Data will be collected at baseline and after the three‑month follow‑up period using a structured questionnaire. The three‑month follow‑up assessment will be conducted during a scheduled clinic appointment at the hospital. At this visit, the same blinded data collectors will re‑administer the full questionnaire. The tool will capture socio‑demographic information, clinical characteristics, and the study outcomes using the following instruments: the General Medication Adherence Scale (GMAS) [39], the Asthma Control Test (ACT) [40], a knowledge assessment questionnaire adopted from previous literature [1,41,42], and a standardized inhaler technique checklist adapted from National Institutes of Health (NIH) guidelines [21]. The original English versions of these instruments will be translated into the local language (Tigrigna) by an independent bilingual translator. This version will be reviewed for content and face validity by an expert panel of senior clinical pharmacists, a pharmacologist, and a chest unit physician. To ensure conceptual accuracy, the Tigrinya version will be back‑translated into English by a different translator, with all discrepancies reconciled. Trained data collectors, blinded to participant group allocation, will administer the tool through face‑to‑face interviews. Inhaler technique will be assessed by direct observation of the participant’s demonstration using their own device, scored against a standardized checklist.

### Statistical analysis

Statistical analysis will be performed using SPSS version 28.0. Descriptive statistics will summarize participant characteristics: categorical variables as frequencies and percentages, continuous variables as means with standard deviations or medians with interquartile ranges. The primary analysis will follow the intention‑to‑treat principle, analyzing all participants according to their original randomized group regardless of whether they completed the intervention. For the primary outcome (dichotomized medication adherence), the proportion of adherent participants will be compared between groups using the chi‑square test. Within‑group changes from baseline to follow‑up will be tested using McNemar test. Between‑group differences at follow‑up will be analyzed using Pearson’s chi‑square test (or Fisher’s exact test when expected cell counts are < 5). For continuous variables with skewed, the Mann‑Whitney U test will be used. Multivariable binary logistic regression will be employed to identify factors associated with poor medication knowledge, medication non‑adherence, and uncontrolled asthma, with the intervention group as the main independent variable adjusted for potential con-founders (age, sex, education, income, Comorbidities, exacerbation history, and smoking status). Effect sizes will be reported as adjusted odds ratios with 95% confidence intervals. Subgroup analyses will be performed to explore whether the intervention effect differs across patient characteristics (age, gender, education, occupation, income, GINA step, comorbidity, exacerbation history) using logistic regression with interaction terms; p‑values for interaction will be reported. Missing data will be handled by complete case analysis if the proportion is low (<5%); otherwise, multiple imputation will be considered. A p‑value < 0.05 will be considered statistically significant.

### Strengths and limitations

This trial will have several strengths, including a randomized controlled design with computer‑generated allocation, blinded outcome assessment, use of validated instruments (GMAS, ACT), and total enumeration of the target population. However, several limitations will be acknowledged. The single‑center design will limit generalizability to other hospitals or primary care settings. The requirement for literacy (or a literate caregiver) and telephone access may introduce selection bias, potentially excluding the most disadvantaged patients. Adherence will be measured by self‑report using the GMAS. The three‑month follow‑up period will not capture long‑term sustainability of the intervention effects.

### Ethical considerations

Ethical approval for this study was obtained from the Mekelle University Institutional Review Board (MU‑IRB; Ref: 2571/2025). Written informed consent will be obtained from all participants prior to any study procedures. For participants who are unable to read, the entire consent form will be read aloud in the presence of an impartial witness, and a thumbprint will be obtained. All methods will be carried out in accordance with relevant guidelines and regulations, including the Declaration of Helsinki, to ensure the protection of participants’ rights, safety, and well‑being.

### Trial status

Participant recruitment for this trial took place from March 1, 2025, to May 31, 2025, and has been completed. The intervention and follow-up phase is currently ongoing. Data collection is active. Data cleaning, coding, and statistical analysis have not yet commenced. The database remains locked and has not been unblinded. No results have been generated, examined, or interpreted.

## Supporting information

S1 Fig Participant timeline – Schedule of enrollment, interventions, and assessments according to SPIRIT 2025 guidelines. (DOCX)

S2 Checklist SPIRIT 2025 checklist – Completed SPIRIT 2013 checklist for trial protocols. (DOCX)

S3 File Data extraction tool (English) – English version of the structured questionnaire used for data collection. (DOCX)

S4 File Data extraction tool (Tigrigna) – Tigrigna version of the structured questionnaire. (DOCX)

S5 File General Medication Adherence Scale (GMAS) – The 11‑item GMAS instrument used to measure medication adherence. (DOCX)

S6 File Asthma Control Test (ACT) – The ACT questionnaire used to assess asthma control. (DOCX)

S7 File Intervention provider guide – Detailed guide for delivering the educational intervention (teach‑back steps, phone call script, SMS content). (DOCX)

S8 File Educational pamphlet (Tigrigna) – Illustrated patient pamphlet used in the intervention (Tigrigna language). (DOCX)

S8 File Educational pamphlet- – Illustrated patient pamphlet used in the intervention (English). (DOCX)
