# Supplementary material for: Pharmacist-led educational intervention to improve knowledge, medication adherence, and asthma control among asthma patients at Ayder Comprehensive Specialized Hospital: A protocol for randomized controlled trial
Source: PLoS One. 2026 Jul 16;21(7):e0349805. doi: 10.1371/journal.pone.0349805 (PMC13375000; doi:10.1371/journal.pone.0349805)
Supplement: S3 File — (DOCX) [file pone.0349805.s003.docx]

**English Version Data Extraction Tool**

**General Characteristics**

Code: ______________

1. Gender: (Please select one)  Male  Female
2. Age: __________
3. Education Level: (Please select one  No formal education  Primary school    Secondary school  Tertiary education and above
4. Occupation: (Please select one)  Student  Farmer  Merchant   employee  Daily laborer Housewives: __________
5. Income (monthly)_______________________

**Clinical Characteristics**

1. Smoking Status:  Smoker  Stopped smoking  Non-smoker
2. Family History of Asthma:  Yes  No
3. Asthma Treatment Step: (select one)  Step 2  Step 3  Step 4  Step 5
4. Comorbidities: (select one)  None  1–2 conditions  3 or more conditions
5. Severe Exacerbations in the Past Year:  None  ≥1

**Section 3 General Medication Adherence Scale (GMAS)**

Please answer the following questions regarding your asthma medication adherence. For each question, select the option that best represents your experience.

1. Do you have difficulty remembering to take your medicine? Never Sometimes  Often  Always
2. Did you forget to take your medicine due to a busy schedule such as travel, meeting, party, wedding, church/temple, etc.? Never  Sometimes  Often  Always
3. Did you stop taking the medicine when you feel well? Never  Sometimes  Often  Always
4. Did you stop taking the medicine when you experienced side effects? Never  Sometimes  Often  Always
5. Did you stop taking your medicine without telling your doctor?  Never  Sometimes  Often  Always
6. Did you stop taking medicine (for asthma) because you have to take more drugs for other diseases? Never  Sometimes  Often  Always
7. Did you find it inconvenient to remember to take your medication because of the complicated regimen? Never  Sometimes  Often  Always
8. In the past month, did you forget to take your medicine because of symptom severity and needed new medicine? Never  Sometimes  Often  Always
9. Did you arbitrarily change the drug regimen, such as dose, and times a day?  Never  Sometimes  Often  Always
10. Did you stop taking your medication because the drugs were not worth the money? Never  Sometimes  Often  Always
11. Did you find it difficult to buy drugs because of their expenses? Never  Sometimes  Often  Always

**Knowledge of Asthma Medications**

1. Can you correctly distinguish between control and reliever medications?

 Correct  Incorrect

1. Do you know how to use the inhalation technique correctly?  Correct  Incorrect

**Asthma Symptom Control Level (In the Past 4 Weeks)**

1. Did you experience more than 2 symptoms a week during the daytime?  Yes  No
2. Did you wake up at night due to asthma?  Yes  No
3. Did you need to use your symptom relief medication more than twice a week? Yes  No
4. Did you experience limitations in your activities due to asthma?  Yes No
