# Supplementary material for: Pharmacist-led educational intervention to improve knowledge, medication adherence, and asthma control among asthma patients at Ayder Comprehensive Specialized Hospital: A protocol for randomized controlled trial
Source: PLoS One. 2026 Jul 16;21(7):e0349805. doi: 10.1371/journal.pone.0349805 (PMC13375000; doi:10.1371/journal.pone.0349805)
Supplement: S4 File — (DOCX) [file pone.0349805.s004.docx]

**Tigrinya Version Data Extraction Tool (መተሓሓዚታት)**

**ሓፈሻዊ ባህርያት**

ኮድ፡ ______________

- - - 1. ጾታ፡ (በጃኹም ሓደ ምረጹ) ተባዕታይ ጓል ኣንስተይቲ
      2. ዕድመ: __________
      3. ደረጃ ትምህርቲ፡ (በጃኹም ሓደ ምረጹ ወግዓዊ ትምህርቲ የለን መባእታ ቤት ትምህርቲ ካልኣይ ደረጃ ትምህርቲ ሳልሳይ ደረጃ ትምህርቲ
      4. ሞያ፡ (በጃኹም ሓደ ምረጹ) ተማሃራይ ሓረስታይ ሰራሕተኛ ነጋዳይ ናይ ብሕቲ ሰራሕተኛ መዓልታዊ ሸቃላይ ካልእ፡ __________
      5. ኣታዊ (ወርሓዊ)_______________________

**ክሊኒካዊ ባህርያት**

- - - 1. ኩነታት ምትካኽ ሽጋራ ፡ ሽጋራ ዘትክኽ ሽጋራ ምትካኽ ኣቋሪጹ ሽጋራ ዘይትክኽ
      2. ስድራቤታዊ ታሪኽ ኣዝማ ፡ እወ ኣይፋልን ።
      3. ሕክምና ኣዝማ ስጉምቲ፡ (በጃኹም ሓደ ምረጹ)ደረጃ 2 ደረጃ 3 ደረጃ 4 ደረጃ 5
      4. ምትሕውዋስ ሕማማት: (ኩሎም ዝምልከቶም ምረጽ) ዝኾነ ይኹን 1–2 ኩነታት 3 ወይ ልዕሊኡ ኩነታት
      5. ኣብ ዝሓለፈ ዓመት ከቢድ ምግዳድ: ዝኾነ ≥1

**ክፍሊ 3 ሓፈሻዊ መለክዒ ምትግባር መድሃኒት (GMAS)**

- - - 1. በጃኹም ነዞም ዝስዕቡ ሕቶታት ብዛዕባ ምኽባር መድሃኒት ኣዝማኹም መልሱ። ንነፍሲ ወከፍ ሕቶ፡ ነቲ ንተመኩሮኻ ብዝበለጸ ዝውክል ኣማራጺ ምረጽ።
      2. ክንደይ ግዜ ኢኻ መድሃኒት ኣዝማኻ ክትወስድ ትርስዕ? ፈጺሙ ሓደ ሓደ ግዜ ብዙሕ ግዜ ኩሉ ግዜ
      3. ክንደይ ግዜ መድሃኒትካ ከምቲ ዝተኣዘዘልካ ትወስዶ? ፈጺሙ ሓደ ሓደ ግዜ ብዙሕ ግዜ ኩሉ ግዜ
      4. ንሓኪምካ ከይተማኸርካ መድሃኒትካ ምውሳድ ኣቋሪጽካ ትፈልጥ ዲኻ? ፈጺሙ ሓደ ሓደ ግዜ ብዙሕ ግዜ ኩሉ ግዜ
      5. ክንደይ ግዜ መድሃኒትካ ኣድላዪ ከምዘይኮነ ኮይኑ ይስምዓካ? ፈጺሙ ሓደ ሓደ ግዜ ብዙሕ ግዜ ኩሉ ግዜ
      6. ክንደይ ግዜ ኢኻ መድሃኒትካ ብኸመይ ከም እትወስዶ ንምርዳእ ትጽገም? ፈጺሙ ሓደ ሓደ ግዜ ብዙሕ ግዜ ኩሉ ግዜ
      7. ክንደይ ግዜ እዩ መድሃኒትካ ምውሳድካ ደው ዘብል ጎናዊ ሳዕቤናት የጋጥመካ? ፈጺሙ ሓደ ሓደ ግዜ ብዙሕ ግዜ ኩሉ ግዜ
      8. ክንደይ ግዜ መድሃኒትካ ምውሳድ ዘይምቹእ ኮይኑ ይስምዓካ? ፈጺሙ ሓደ ሓደ ግዜ ብዙሕ ግዜ ኩሉ ግዜ
      9. ክንደይ ግዜ መድሃኒትካ ክትወስድ ክትዝክር ትሽገር? ፈጺሙ ሓደ ሓደ ግዜ ብዙሕ ግዜ ኩሉ ግዜ
      10. ክንደይ ግዜ ኢኻ ብብዝሒ ክትወስዶ ዘለካ መድሃኒታት ልዕሊ ዓቕምኻ ዝስምዓካ?

ፈጺሙ ሓደ ሓደ ግዜ ብዙሕ ግዜ ኩሉ ግዜ

- - - 1. ክንደይ ግዜ ምስ ወሃቢ ክንክን ጥዕናኻ ብዛዕባ ሕክምና ኣዝማ ትዘራረብ? ፈጺሙ ሓደ ሓደ ግዜ ብዙሕ ግዜ ኩሉ ግዜ
      2. ክንደይ ግዜ መድሃኒታት ኣዝማኻ ኣብ ምምሕዳር ርእሰ ተኣማንነት ይስምዓካ? ፈጺሙ ሓደ ሓደ ግዜ ብዙሕ ግዜ ኩሉ ግዜ
      3. **ፍልጠት መድሃኒታት ኣዝማ**
      4. ኣብ መንጎ ንመቆጻጸርን መሕወዪን መድሃኒታት ብትኽክል ክትፈልዮም ትኽእል ዲኻ?ቅኑዕ ጌጋ
      5. ነቲ ናይ ምትንፋስ ሜላ ብትኽክል ከመይ ጌርካ ከም እትጥቀመሉ ትፈልጥ ዲኻ?ቅኑዕ ጌጋ
      6. **ደረጃ ምቁጽጻር ምልክታት ኣዝማ (ኣብ ዝሓለፉ 4 ሰሙናት)**
      7. ኣብ ሰሙን ልዕሊ 2 ምልክታት ኣብ መዓልቲ ኣጋጢሙካ ድዩ? እወ ኣይፋልን።
      8. ብሰንኪ ኣዝማ ምሸት ዲኻ ተበራቢርካ? እወ ኣይፋልን።
      9. ኣብ ሰሙን ልዕሊ ክልተ ግዜ ምልክታትካ ዘቃልል መድሃኒት ክትጥቀመሉ ኣድለየካ ድዩ? እወ ኣይፋልን።
      10. ብሰንኪ ኣዝማ ኣብ ንጥፈታትካ ድሩትነት ኣጋጢሙካ ድዩ? እወ ኣይፋልን።
