# Supplementary material for: Pharmacist-led educational intervention to improve knowledge, medication adherence, and asthma control among asthma patients at Ayder Comprehensive Specialized Hospital: A protocol for randomized controlled trial
Source: PLoS One. 2026 Jul 16;21(7):e0349805. doi: 10.1371/journal.pone.0349805 (PMC13375000; doi:10.1371/journal.pone.0349805)
Supplement: S5 File — (DOCX) [file pone.0349805.s005.docx]

**General Medication Adherence Scale**

**Table 5 General Medication Adherence Scale (GMAS)**

|  | **Question Description** | **Score (0-3)** |
| --- | --- | --- |
| 1 | Do you have difficulty remembering to take your medicine? | 0 = Always  1 = Often  2 = Sometimes  3 = Never |
| 2 | Did you forget to take your medicine due to a busy schedule (e.g., travel, meetings, events)? |  |
| 3 | Did you stop taking the medicine when you feel well? |  |
| 4 | Did you stop taking the medicine when you experienced side effects (e.g., stomach pain)? |  |
| 5 | Did you stop taking your medicine without telling your doctor? |  |
| 6 | Did you stop taking medicine (for asthma) because you have to take more drugs for other diseases? |  |
| 7 | Did you find it inconvenient to remember to take your medication because of the complicated regimen? |  |
| 8 | In the past month, did you forget to take your medicine because of symptom severity and needed new medicine? |  |
| 9 | Did you arbitrarily change the drug regimen (e.g., dose, frequency)? |  |
| 10 | Did you stop taking your medication because the drugs were not worth the money? |  |
| 11 | Did you find it difficult to buy drugs because of their expenses? |  |
|  | **Adherence Levels**: ***High adherence***: Total score of 30–33 points, ***good adherence***: Total score of 27–29 points**, *Partial adherence***: Total score of 17–26 points, ***Low adherence***: Total score of 11–16 points and ***Poor adherence***: Total score of 0–10 points  **>= 27 Adherence <=26 non-Adherence** | |
