# Supplementary material for: Pharmacist-led educational intervention to improve knowledge, medication adherence, and asthma control among asthma patients at Ayder Comprehensive Specialized Hospital: A protocol for randomized controlled trial
Source: PLoS One. 2026 Jul 16;21(7):e0349805. doi: 10.1371/journal.pone.0349805 (PMC13375000; doi:10.1371/journal.pone.0349805)
Supplement: S6 File — (DOCX) [file pone.0349805.s006.docx]

**Asthma Symptom Control Level**

| In the Past 4 Weeks | |
| --- | --- |
| Did you experience more than 2 symptoms a week during the daytime?  Yes  No | Uncontrolled (3-4)  Partly Controlled (1-2)  Well Controlled (0)  Yes=1, No= 0 |
| Did you wake up at night due to asthma?  Yes No |  |
| Did you need to use your symptom relief medication more than twice a week?  Yes No |  |
| Did you experience limitations in your activities due to asthma?  Yes No |  |
