# Supplementary material for: Pharmacist-led educational intervention to improve knowledge, medication adherence, and asthma control among asthma patients at Ayder Comprehensive Specialized Hospital: A protocol for randomized controlled trial
Source: PLoS One. 2026 Jul 16;21(7):e0349805. doi: 10.1371/journal.pone.0349805 (PMC13375000; doi:10.1371/journal.pone.0349805)
Supplement: S7 File — (DOCX) [file pone.0349805.s007.docx]

## Interviewer Guide for Face-to-Face Education and Counseling

**Interviewer Guide for Face-to-Face Education and Counseling**

**Introduction:** This guide is designed to help you deliver personalized educational sessions to asthmatic patients. The goal is to promote medication adherence and improve overall asthma control by providing clear and practical information.

**Contact Information:** If participants have any questions or need further assistance, please direct them to contact us at: Phone: 0910551098 / 0715558335

**1. Understanding Asthma Pathophysiology and Its Triggers:**

**Explanation:**

- "Asthma is a chronic condition that affects your airways, causing them to become inflamed and narrow. This makes it hard for air to move in and out of your lungs, leading to symptoms like wheezing, coughing, chest tightness, and shortness of breath."

**Triggers:**

- "Asthma can be triggered by various factors. Common triggers include:
  - **Allergens:** Pollen, dust mites, pet dander, mold.
  - **Irritants:** Smoke, strong odors, pollution.
  - **Infections:** Cold, flu, respiratory infections.
  - **Physical Activity:** Exercise.
  - **Weather Conditions:** Cold air, sudden changes in weather. Identifying your specific triggers can help you avoid them and manage your asthma better."

**2. The Importance of Adhering to Prescribed Medications:**

**Medication Necessity:**

- "Taking your medications as prescribed by your doctor is crucial for managing asthma. Even if you feel well, continuing your medication helps keep your asthma under control and prevents future attacks."

**Types of Medications:**

- "There are two main types of asthma medications:
  - **Controller Medications:** These are taken daily to prevent symptoms and reduce inflammation. Example: Inhaled corticosteroids.
  - **Reliever Medications:** These provide quick relief from symptoms during an asthma attack. Example: Short-acting beta-agonists."

**3. Proper Inhaler Techniques:**

**Demonstration:**

- "Let's go over the correct way to use your inhaler:

**The steps to use the asthma medication are as follows:**

- 1. Take the cap off the inhaler.
- 2. Shake the inhaler hard 10 to 15 times before each use.
- 3. Breathe out fully, trying to push out as much air as possible.
- 4. Hold the inhaler with the mouthpiece down and place your lips around it to form a tight seal.
- 5. As you start to slowly breathe in through your mouth, press down on the inhaler once.
- 6. Keep breathing in slowly and deeply.
- 7. Wait about 1 minute before taking the next puff.
- 8. Replace the cap on the mouthpiece and ensure it is firmly closed.

**Practice:**

- "Please demonstrate how you use your inhaler to ensure you are using it correctly. I will provide feedback and guidance as needed."

**4. Recognition of Potential Side Effects of Medications:**

**Common Side Effects:**

- "Some common side effects of asthma medications include:
  - **Inhaled Corticosteroids:** Throat irritation, hoarseness, oral thrush.
  - **Short-Acting Beta-Agonists:** Jitteriness, rapid heartbeat."

**Management Strategies:**

- "To manage side effects, you can:
  - **Rinse Your Mouth:** Rinse your mouth with water after using your inhaler to reduce the risk of throat irritation and oral thrush.
  - **Consult Your Doctor:** Speak with your doctor about any side effects you experience. They can adjust your treatment if necessary."

**5. Lifestyle Modifications:**

**Avoiding Triggers:**

- "Making some lifestyle changes can help prevent asthma exacerbations:
  - **Use Allergy-Proof Covers:** Protect your pillows and mattresses from dust mites.
  - **Keep Pets Out of the Bedroom:** Reduce exposure to pet dander.
  - **Use Air Purifiers:** Clean the air in your home to reduce smoke and pollution.
  - **Stay Indoors During High Pollen Days:** Minimize exposure to outdoor allergens."

**Healthy Habits:**

- "Maintaining a healthy lifestyle can improve your overall health and asthma control:
  - **Healthy Diet:** Eat a balanced diet rich in fruits and vegetables.
  - **Regular Exercise:** Engage in physical activity that is appropriate for your condition.
  - **Good Sleep Hygiene:** Ensure you get enough restful sleep."

**6. Strategies to Enhance Adherence:**

**Set Reminders:**

- "Use tools like phone alarms, medication reminder apps, or calendar alerts to help you remember to take your medication."

**Develop a Routine:**

- "Incorporate medication-taking into your daily routine by linking it to another regular activity, such as brushing your teeth."

**Support Systems:**

- "Involve family members or friends in your medication routine. They can provide support and help remind you to take your medication.
