# Supplementary material for: Pharmacist-led educational intervention to improve knowledge, medication adherence, and asthma control among asthma patients at Ayder Comprehensive Specialized Hospital: A protocol for randomized controlled trial
Source: PLoS One. 2026 Jul 16;21(7):e0349805. doi: 10.1371/journal.pone.0349805 (PMC13375000; doi:10.1371/journal.pone.0349805)
Supplement: S8 File — (DOCX) [file pone.0349805.s008.docx]

## ትምህርታዊ በራሪ ፅሑፍ

**ትምህርታዊ በራሪ ፅሑፍ**

**እንታይነት ሕማም ኣስሚ**

**ሕማም ኣስሚ እንታይ እዩ** ? ሕማም ኣስሚ  ንስርዓተ ምትንፋስ ኣፀጋሚ ክኸውን ዝገብር ኩነት እዩ። ትቦታት መተሓላለፊ ኣየር ስለ ዝሓብጡን  ዝፀብቡን፥  ምስሓግ (ሕፂፅ ዝብል ድምፂ ምህላው/ምንኣኽ)፣ ምስዓል፣ ምዕፋን ኣፍልቢን ምሕፃር ምትንፋስን የስዕብ።

**መልዓሊ ምኽንያት ሕማም ኣስሚ እንታይ እዩ ?** ጽገ፣ደሮና ፣ትኪ፣ዝሑል ኣየርን ኣካላዊ ምንቅስቓስን ዝኣመሰሉን ኣስሚ ከለዓዕሉ ዝኽእሉ ምኽንያታት እዮም። እንታይነትን ዓይነትን መለዓዓሊ ምኽንያት ምፍላጥ ካብኡ ንምርሓቕን ነቲ ሕማም ብዝሓሸ መገዲ ንምቁፅፃር ኣዝዩ ሓጋዚ እዩ ።

ንኣስሚ ምቁፅፃር፦

• መድሓኒትካ/ኪ ብግቡእ ውሰድ/ዲ

• ንመተንፈሲ መሳርሒታትካ/ኪ ብግቡእ ተጠቐመሉ/ምሉ፦

• ንሕማም መለዓዓልቲ ነገራትን ኩነታትን ኣወግድ/ዲ፦

• ስሩዕ ክትትል ምርመራ ምግባር፦

ግቡእ ኣወሳስዳ መድሓኒት፦ ዝሓሸ ምቁፅፃር ኣስማ ንምርካብ ዝሕግዝ ዋና መፍትሒ እዩ

መዓልታዊ መድሓኒት ኣስሚ ምውሳድ እዞም ዝስዕቡ ጥቕምታት ኣለዎ፦

• ምስትንፋስ ቀሊል ይኸውን

• ሃንደበታዊ መጥቃዕቲ ኣስሚ ውሑድ ክኸውን ይገብር

• ካብ ብተደጋጋሚ ሆስፒታል ምድቃስ የርሕቕ

• ዝሓሸ ህይወት ይህልው

መድሓኒት ኣብ ምውሳድ ዘጋጥሙ ልሙዳት ጸገማት፦

•መድሓኒት ምውሳድ ምርሳዕ

•ብዛዕባ ጎናዊ ሳዕቤናት ምጭናቕ

•ኣድላይነት መድሓኒት ዘይምርዳእ

•ምኽባር ዋጋ  መድሓኒት

መድሓኒት ዝውሰደሉ ሰዓት ብኸመይ ምዝካር ይከኣል ፦

• ኣብ ስልክኻ/ኺ መዓልታዊ መዘኻኸሪታት ምምላእ።

•ንመድሓኒትካ/ኪ ኽትርእዮ ኣብ እትኽእለሉ ቦታ ምቕማጥ።

•ብዛዕባ ዝዀነ ይኹን ዘተሓሳስበካ/ኪ ነገር ንሓኪምካ/ኪ ምዝርራብ።

ቅደም ሰዓብ ኣጠቓቕማ መተንፈሲ መሳርሒ፦

1. ነቲ መኽደን እቲ መተንፈሲ መሳርሒ ኣውፃኣዮ/እዮ።

2.ቕድሚ እቲ መሳርሒ ምጥቃምካ/ኪ ካብ 10 ኽሳዕ 15 ሳዕ ምሕቛን።

3. ብእተኻእለካ/ኪ መጠን  ንደገ ብምስትንፋስ ኣየር ንክወፅእ ምግባር ።

4. ኣፍ እቲ መተንፈሲ መሳርሒ ንታሕቲ ገፅ ሓዞ/ዝዮ እሞ ናብ ውሽጢ ከናፍርካ/ኪ ኣእቲኻ/ኺ ቀርቅሮ/ርዮ።

5.ቀስ ብቐስ ብኣፍካ  ንውሽጢ ክተስተንፍስ ምስ ጀመርካ/ኪ ሓደ ጊዜ ነቲ መተንፈሲ መሳርሒ ጸቕጢ ግበረሉ/ርሉ።

6. ቀስ ኢልካን/ኪኔ ኣዕሚቝካን/ኪን ንውሽጢ ምስትንፋስካ/ኪ ቀፅል/ሊ ።

7. ቀፂልካ ቅድሚ ምንፋሕኻ/ኺ 1 ደቒቕ ዝኸውን ተጸበ/በዪ።

8. ኣፍ እቲ መተንፈሲ መሳርሒ ክደኖ/ንዮ እሞ ብጽኑዕ ከም እተኸደነ ኣረጋግፅ/ፂ።

ንሃንደበታዊ መጥቃዕቲ ቅድመ ምድላው፦

• ኩልጊዜ ነቲ መተንፍሲ መሳርሒኻ/ኺ ሓዞ/ዝዮ።

• ናይ መጀመርታ ምልክታት ምብእኣስ ሕማም ኣስማ ምፍላጥ።

ንመጥቃዕቲ ኣስሚ ፈሊኻ ንምፍላጥ፦

•  ከቢድ ፀገም ስርዓተ ምስትንፋስ ይህሉ

• ቀፃላይ ዝኾነ ምስሓግ ወይ ምስዓል ይህሉ

• ምዕባስ ኣፍ ልቢ ይህሉ

• ምዝራብ ይኸብደካ/ኪ

ኣብ እዋን መጥቃዕቲ  ክትገብሮም/ዮም ዝግብኡ ነገራት፦

1. ነቲ መተንሰሲ መሳርሒኻ ብኡንብኡ(ወድያው) ምጥቃም።

2. ኮፍ ምባልን ምህዳእን።

3. ነቲ ኣቐዲምካ ንክትገብሮ ዝወጠንካዮ  መደብ ኣተኣላልያ ኣስሚ ስዓቦ።

4. እንተ ዘይተመሓየሸ ብቕልጡፍ ሕክምናዊ ሓገዝ ምርካብ።
