# Supplementary material for: Pharmacist-led educational intervention to improve knowledge, medication adherence, and asthma control among asthma patients at Ayder Comprehensive Specialized Hospital: A protocol for randomized controlled trial
Source: PLoS One. 2026 Jul 16;21(7):e0349805. doi: 10.1371/journal.pone.0349805 (PMC13375000; doi:10.1371/journal.pone.0349805)
Supplement: S8 File — (DOCX) [file pone.0349805.s009.docx]

**Educational Brochure**

**Introduction:** This brochure aims to provide clear, simple information for asthmatic patients on managing their condition effectively. It includes essential topics such as understanding asthma, the importance of medication adherence, correct inhaler techniques, managing triggers, and emergency management. By offering easy-to-understand guidelines and practical tips, the brochure helps patients enhance their knowledge and skills, leading to better asthma control and improved quality of life.

**Understanding Asthma**

**What is Asthma?** Asthma is a condition that makes it hard to breathe. Your airways become swollen and narrow, causing wheezing, coughing, chest tightness, and shortness of breath.

**What Causes Asthma?** Asthma can be triggered by things like pollen, dust, smoke, cold air, and exercise. Identifying what triggers your asthma can help you avoid them and manage your condition better.

**Managing Asthma:**

- **Take Your Medicine:** Always take your medicine as your doctor told you, even when you feel fine.
- **Use Inhalers Correctly:** Make sure you know how to use your inhaler properly.
- **Avoid Triggers:** Stay away from things that trigger your asthma.
- **Regular Check-ups:** Visit your doctor regularly to keep your asthma under control.

**Medication Adherence: Key to Better Asthma Control**

**Why Take Your Medicine?** Taking your asthma medicine every day helps you:

- Breathe easier
- Have fewer asthma attacks
- Stay out of the hospital
- Enjoy a better quality of life

**Common Problems with Taking Medicine:**

- Forgetting to take it
- Worried about side effects
- Not understanding why it's needed
- Cost of the medicine

**How to Remember to Take Your Medicine:**

- Set daily reminders on your phone.
- Keep your medicine where you can see it.
- Talk to your doctor about any concerns.

**The steps to use the asthma medication are as follows:**

- 1. Take the cap off the inhaler.
- 2. Shake the inhaler hard 10 to 15 times before each use.
- 3. Breathe out fully, trying to push out as much air as possible.
- 4. Hold the inhaler with the mouthpiece down and place your lips around it to form a tight seal.
- 5. As you start to slowly breathe in through your mouth, press down on the inhaler once.
- 6. Keep breathing in slowly and deeply.
- 7. Wait about 1 minute before taking the next puff.
- 8. Replace the cap on the mouthpiece and ensure it is firmly closed.

**Managing Asthma Triggers**

**What Triggers Asthma?**

- **Allergens:** Pollen, dust mites, pets, mold
- **Irritants:** Smoke, pollution, strong smells
- **Infections:** Cold, flu
- **Exercise:** Physical activity
- **Weather:** Cold air, weather changes

**How to Avoid Triggers:**

- Use covers on pillows and mattresses to protect against dust mites.
- Keep pets out of the bedroom.
- Use air purifiers to reduce smoke and pollution.
- Get vaccinated to protect against infections.
- Use your inhaler before exercising.
- Wear a scarf over your mouth and nose in cold weather.

**Emergency Preparedness:**

- Always carry your inhaler.
- Know the early signs of an asthma attack.
- Follow your asthma action plan during an attack.

**Emergency Management of Asthma**

**Recognizing an Asthma Attack:**

- Severe trouble breathing
- Constant wheezing or coughing
- Tight feeling in the chest
- Difficulty speaking

**What to Do During an Attack:**

1. Use your inhaler right away.
2. Sit up and stay calm.
3. Follow your asthma action plan.
4. If it doesn’t get better, get medical help fast.

**After an Attack:**

- See your doctor to review your plan.
- Make sure you have enough medicine.
- Talk to your doctor about how to prevent future attacks.

**Contact Information:** If you have any questions or need further assistance, please get in touch with us at: Phone: 0910551098 / 0715558335
