# Supplementary material for: Pharmacist-led educational intervention to improve knowledge, medication adherence, and asthma control among asthma patients at Ayder Comprehensive Specialized Hospital: A protocol for randomized controlled trial
Source: PLoS One. 2026 Jul 16;21(7):e0349805. doi: 10.1371/journal.pone.0349805 (PMC13375000; doi:10.1371/journal.pone.0349805)
Supplement: Supplementary file 9 [file pone.0349805.s001.docx]

TIMEPOINT	Enrollment	Post-randomization			Close-out
	**-t1**	**0**	**t1**	**t2**	**t3**
**ENROLLMENT:**					
Eligibility Screen	X				
Informed Consent	X				
**ALLOCATION:**					
Randomization		X			
**INTERVENTION/ COMPARATOR:**					
Intervention Group (Enhanced Counselling)		X			
· Follow-up Phone Call			X		
· Follow-up Text Message				X	
Control Group (Standard Care)		X	X	X	X
**ASSESSMENTS:**					
Demographics & Clinical History		X			
Knowledge Assessment Questionnaire		X			X
General Medication Adherence Scale (GMAS)		X			X
Asthma Control Test (ACT)		X			X
Adverse Event Monitoring		X	X		

t1: Follow-up phone call, **t2:** Follow-up text message, **t3 (Close-out):** Final assessment

Figure 1 Figure 1 Participant Timeline: Schedule of Enrollment, Interventions, and Assessment
